# Clinical Utility of the 12-Gene DCIS Score Assay: Impact on Radiotherapy Recommendations for Patients with Ductal Carcinoma In Situ

**DOI:** 10.1245/s10434-016-5583-7

**Published:** 2016-10-04

**Authors:** Jennifer B. Manders, Henry M. Kuerer, Benjamin D. Smith, Cornelia McCluskey, William B. Farrar, Thomas G. Frazier, Linna Li, Charles E. Leonard, Dennis L. Carter, Sheema Chawla, Lori E. Medeiros, J. Michael Guenther, Lauren E. Castellini, Daniel J. Buchholz, Eleftherios P. Mamounas, Irene L. Wapnir, Kathleen C. Horst, Anees Chagpar, Suzanne B. Evans, Adam I. Riker, Faisal S. Vali, Lawrence J. Solin, Lisa Jablon, Abram Recht, Ranjna Sharma, Ruixiao Lu, Amy P. Sing, E. Shelley Hwang, Julia White, Study investigators and study participants

**Affiliations:** 1 The Christ Hospital Health Network, Cincinnati, OH USA; 2 University of Texas MD Anderson Cancer Center, Houston, TX USA; 3 Ohio State University James Cancer Hospital, Columbus, OH USA; 4 Bryn Mawr Hospital, Bryn Mawr, PA USA; 5 Rocky Mountain Cancer Centers, Denver, CO USA; 6 Rochester Regional Health System, Rochester, NY USA; 7 Saint Elizabeth Medical Center, Inc., Edgewood, KY USA; 8 UF Health Cancer Center at Orlando Health, Orlando, FL USA; 9 Stanford Cancer Institute, Stanford University, Palo Alto, CA USA; 10 Yale University, New Haven, CT USA; 11 Advocate Christ Medical Center, Oak Lawn, IL USA; 12 Albert Einstein Healthcare Network, Philadelphia, PA USA; 13 Beth Israel Deaconess Medical Center, Boston, MA USA; 14 Genomic Health, Inc., Redwood City, CA USA; 15 Duke University Medical Center, Durham, NC USA; 16 Louisiana State University Health New Orleans, New Orleans, LA USA

## Abstract

**Objective:**

The aim of this study was to determine the impact of the results of the 12-gene DCIS Score assay on (i) radiotherapy recommendations for patients with pure ductal carcinoma in situ (DCIS) following breast-conserving surgery (BCS), and (ii) patient decisional conflict and state anxiety.

**Methods:**

Thirteen sites across the US enrolled patients (March 2014–August 2015) with pure DCIS undergoing BCS. Prospectively collected data included clinicopathologic factors, physician estimates of local recurrence risk, DCIS Score results, and pre-/post-assay radiotherapy recommendations for each patient made by a surgeon and a radiation oncologist. Patients completed pre-/post-assay decisional conflict scale and state-trait anxiety inventory instruments.

**Results:**

The analysis cohort included 127 patients: median age 60 years, 80 % postmenopausal, median size 8 mm (39 % ≤5 mm), 70 % grade 1/2, 88 % estrogen receptor-positive, 75 % progesterone receptor-positive, 54 % with comedo necrosis, and 18 % multifocal. Sixty-six percent of patients had low DCIS Score results, 20 % had intermediate DCIS Score results, and 14 % had high DCIS Score results; the median result was 21 (range 0–84). Pre-assay, surgeons and radiation oncologists recommended radiotherapy for 70.9 and 72.4 % of patients, respectively. Post-assay, 26.4 % of overall recommendations changed, including 30.7 and 22.0 % of recommendations by surgeons and radiation oncologists, respectively. Among patients with confirmed completed questionnaires (*n* = 32), decision conflict (*p* = 0.004) and state anxiety (*p* = 0.042) decreased significantly from pre- to post-assay.

**Conclusions:**

Individualized risk estimates from the DCIS Score assay provide valuable information to physicians and patients. Post-assay, in response to DCIS Score results, surgeons changed treatment recommendations more often than radiation oncologists. Further investigation is needed to better understand how such treatment changes may affect clinical outcomes.

**Electronic supplementary material:**

The online version of this article (doi:10.1245/s10434-016-5583-7) contains supplementary material, which is available to authorized users.

Ductal carcinoma in situ (DCIS) comprises a heterogeneous group of neoplastic lesions confined to the breast ducts. The incidence of DCIS has increased dramatically over the last quarter-century, since mammography in most women aged 40 years and older became routine.^1^ DCIS now accounts for nearly 18 % of all breast cancers diagnosed in the US.^2^

The current treatment paradigm for DCIS involves initial mastectomy or lumpectomy [also known as breast-conserving surgery (BCS)], often with radiotherapy following lumpectomy and endocrine therapy for patients with hormone receptor-positive disease.^3^ Rates of ipsilateral or local recurrence after BCS alone can range from 14 to 60 % at 10 years, and the addition of radiotherapy reduces the risk by at least half.^4^–^9^ None of the randomized trials comparing BCS alone against BCS with radiotherapy has identified a patient subgroup that did not experience a reduction in the risk of local recurrence with radiotherapy after BCS, nor has there been a change in disease-specific or overall survival from adding radiotherapy detected in any of the trials.^4^,^10^

A clinical conundrum now exists for patients diagnosed with DCIS: against the backdrop of increased detection of DCIS and reduced recurrence risk with BCS plus radiotherapy, there has been little change over time in the incidence of invasive disease related to DCIS or the rate of breast cancer-related death with BCS plus radiotherapy in patients with DCIS.^11^ The increased incidence of DCIS stemming from the adoption of widespread mammographic screening has included proportionally more cases with less adverse clinicopathologic features.^12^ Two clinical trials conducted in this more modern DCIS cohort have revealed relatively lower event rates for recurrence post-lumpectomy, either with or without radiotherapy.^5^,^13^ This supports then that patients with DCIS undergoing BCS do not benefit uniformly from radiotherapy and therefore the treatment approach may represent overtreatment (i.e. the risks of radiotherapy outweigh the benefits) for many in current practice. There is no means to reliably identify individual patients most likely to have a recurrence, who would meaningfully benefit from added radiotherapy.

Traditional approaches to selecting patients for radiotherapy after surgical excision rely on clinicopathologic factors, such as patient age, tumor size, tumor grade, and margin width, to estimate local recurrence risk. Risk estimates based on clinicopathologic factors represent averages derived from population studies and lack precision when applied to individual patients.^14^

The Oncotype DX^®^ Breast DCIS Score™ assay is the first multigene assay that provides independent, individualized estimates of 10 year risk of any local recurrence (DCIS or invasive) and invasive local recurrence. The DCIS Score assay has been clinically validated in two independent studies of 898 patients who had BCS alone, which showed that the DCIS Score result stratifies patients into risk groups, based on the expression of 12 genes (seven cancer-related genes and five reference genes).^15^,^16^

In the first study to evaluate clinical utility of the DCIS Score assay, radiotherapy recommendations changed 31.3 % of the time after the assay results were known.^17^ Clinical utility and similar rates of change in treatment recommendations have also been reported for Oncotype DX genomic assays in invasive breast cancer (35 %) and stage II colon cancer (29 %).^18^,^19^ The consistency of the change rate across diverse tumor types suggests that this magnitude of change is clinically meaningful.

This second clinical utility study of the DCIS Score assay assessed the impact of DCIS Score results on the recommendations made by surgeons and radiation oncologists regarding radiotherapy use, in order to gain insight into assay utility overall and by specialty. This study also investigated the effect of DCIS Score results on the degree of decisional conflict and anxiety that patients felt about their treatment decisions.

## Methods

### Study Design

This was a prospectively enrolled study with the primary objective of determining the impact of the DCIS Score assay on radiotherapy recommendations for patients with DCIS who have undergone BCS. A surgeon and a radiation oncologist independently completed pre- and post-assay questionnaires about their recommendations for each patient. Secondary objectives were to summarize patient clinicopathologic characteristics, to determine the distribution of DCIS Score results across the cohort and across clinicopathologic factors, and to evaluate the effect of DCIS Score results on physician estimates of local recurrence risk. An exploratory objective was to assess changes in patient decisional conflict and anxiety using validated tools, pre- and post-assay. The study was approved by the Institutional Review Board at each site.

### Patient Population

Eligible patients were aged ≥18 years, had histologically confirmed pure DCIS, were candidates for BCS, and were naïve to radiotherapy. Patients with lobular carcinoma in situ, Paget’s disease, known BRCA 1/2 mutation, multicentric disease, invasive carcinoma, or contraindications to radiotherapy were excluded from the study. All enrolled patients provided signed informed consent. The number of patients enrolled met accrual targets.

### Assessments and Tools

Questionnaires captured physicians’ estimates of local recurrence risk, physicians’ treatment recommendations, and factors affecting these recommendations. Pre-assay questionnaires were completed before the assay was ordered, while post-assay questionnaires were completed after the DCIS Score results were available. Clinical and pathologic characteristics were collected for each patient at the time of enrollment at the treating institution; there was no central pathology review. Margin width distances were determined by the treating physician at each institution. The decisional conflict scale (DCS; 16 items) was used to assess patient perceptions of personal uncertainty in making healthcare treatment decisions, and patient satisfaction with treatment decision making.^20^ The state-trait anxiety inventory (STAI; 40 items) was used to measure state anxiety (i.e. anxiety at the moment) and trait anxiety (i.e. overall disposition with respect to anxiety).^21^ Patient participation in completing these instruments was optional.

### Oncotype DX Breast DCIS Score Assay

All DCIS Score assays were performed at Genomic Health, Inc. (GHI; Redwood City, CA, USA) on surgical excision or core biopsy specimens, as previously described.^15^ After assay completion, GHI returned DCIS Score results to the ordering physicians; DCIS Score results are reported on a scale from 0 to100. Risk categories are based on predefined cut-offs: low (<39), intermediate (39–54), and high (≥55).^22^

### Statistical Methods

Changes in radiotherapy recommendations made by surgeons, radiation oncologists, and both specialties combined were described using frequencies, percentages and exact 95 % confidence intervals (CIs). The distribution of DCIS Score results across clinicopathologic factors was summarized using descriptive statistics. Pre- and post-assay 10 year estimates of local recurrence risk were summarized by specialty and for both specialties combined. All hypothesis tests were two-sided and a *p* value < 0.05 was considered statistically significant. All CIs were two-sided 95 % CIs, and CIs for percentages used the exact (Clopper-Pearson) method. Mean changes in the DCS score and STAI scores (S-anxiety score and T-anxiety score) from baseline to follow-up were calculated using 95 % CIs; paired-sample *t*-tests were used to determine whether changes were significantly different from zero. All analyses were conducted using SAS^®^ version 9.4 (SAS Institute, Cary, NC, USA).

## Results

### Patients

At 13 sites across the US, 27 surgeons and 27 radiation oncologists altogether enrolled 141 patients from March 2014 to August 2015. Of these, 127 patients were evaluable. The remaining 14 patients were excluded from analyses because of insufficient tumor (7), insufficient RNA extracted (2), post-assay treatment decision made by the patient without knowledge of the DCIS Score result (2), pre-assay loss to follow-up (1), pre-assay withdrawal of consent (1), and pre-assay patient case report form not completed within the expected timeframe (1). Clinicopathologic characteristics of the evaluable cohort are summarized in Table 1.

**Table 1:** Baseline patient and tumor characteristics of evaluable patients

Characteristic	Category	All evaluable patients^a^ (*n* = 127)
Age, years	Median (range)	60 (34–83)
	<50	19 (15.0)
	50–59	43 (33.9)
	≥60 years	65 (51.2)
Menopausal status	Postmenopausal	101 (79.5)
	Premenopausal	26 (20.5)
DCIS grade	Low	27 (21.3)
	Intermediate	62 (48.8)
	High	38 (29.9)
Margin status	Positive	5 (3.9)
	Negative	121 (95.3)
	Uncertain	1 (0.8)
Margin width, mm	Median (range)	3 (0–32)
	<1	12 (9.4)
	1–3	58 (45.7)
	>3–<5	9 (7.1)
	≥5–<10	22 (17.3)
	≥10	20 (15.7)
Tumor size, mm	Mean (SD)	11.1 (10.2)
	Median (range)	8.0 (1.1–54.0)
	≤5	49 (38.6)
	>5–≤10	26 (20.5)
	>10–≤20	39 (30.7)
	>20	12 (9.4)
Multifocal within excisional specimen	No	104 (81.9)
	Yes	23 (18.1)
DCIS pattern	Solid	56 (44.1)
	Cribriform	52 (40.9)
	Micropapillary	8 (6.3)
	Papillary	5 (3.9)
	Missing	6 (4.7)
Comedo necrosis	Absent	50 (39.4)
	Present	68 (53.5)
	Unknown	9 (7.1)
ER by IHC	Positive	
	All positive	113 (89.0)
	1–10 %	4 (3.1)
	>10 %	105 (82.7)
	Unknown	4 (3.1)
	Negative	12 (9.4)
	Unknown/not done	2 (1.6)
ER by RT-PCR	Positive	115 (90.6)
	Negative	12 (9.4)
PR by IHC	Positive	99 (78.0)
	Negative	22 (17.3)
	Unknown/not done	6 (4.7)
PR by RT-PCR	Positive	100 (78.7)
	Negative	27 (21.3)

Data are expressed as *n* (%) unless otherwise specified

*DCIS* ductal carcinoma in situ, *ER* estrogen receptor, *IHC* immunohistochemistry, *PR* progesterone receptor, *RT*-*PCR* reverse transcription-polymerase chain reaction, *SD* standard deviation

^a^ Fourteen patients were enrolled but were not evaluable as a result of a pathology issue (insufficient tumor; *n* = 7), pre-assay deviation (*n* = 3), laboratory issue (insufficient RNA extracted; *n* = 2), and post-assay deviation (*n* = 2)

### Distribution of DCIS Score Results

**Fig. 1:**
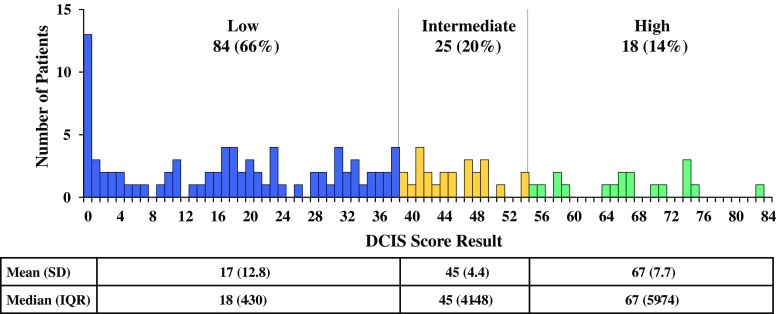
Distribution of DCIS Score results by risk group. The number of patients with DCIS Score results in the low (*blue*), intermediate (*yellow*), and high (*green*) ranges are reported, along with mean (SD) and median (IQR) DCIS Score results for each risk group. *DCIS* ductal carcinoma in situ, *SD* standard deviation, *IQR* interquartile range

**Fig. 2:**
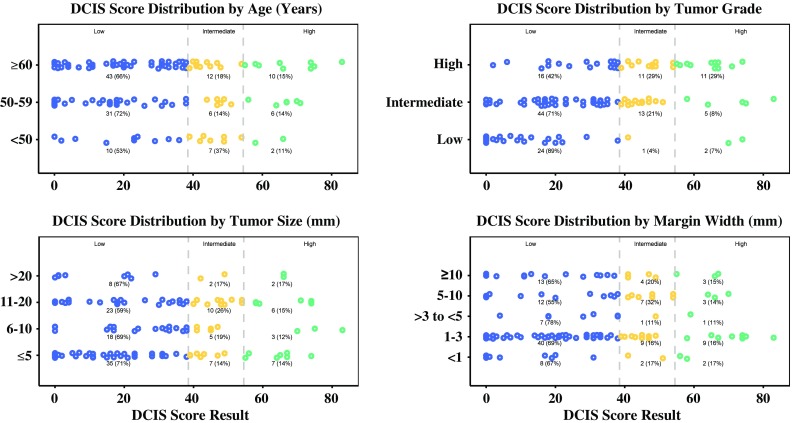
Distribution of DCIS Score results by clinicopathologic factors. The number (%) of patients with DCIS Score results distributed across the low (*blue*), intermediate (*yellow*), and high (*green*) ranges are shown in *dot plots*, subcategorized by age, tumor grade, tumor size, and margin widths. *DCIS* ductal carcinoma in situ

Of 127 evaluable patients, 84 (66 %) had low DCIS Score results, 25 (20 %) had intermediate DCIS Score results, and 18 (14 %) had high DCIS Score results (Fig. 1). A broad distribution of DCIS Score results was evident within each category of age, tumor grade, tumor size, and margin width (Fig. 2).

### Changes in Radiation Treatment Recommendations

**Fig. 3:**
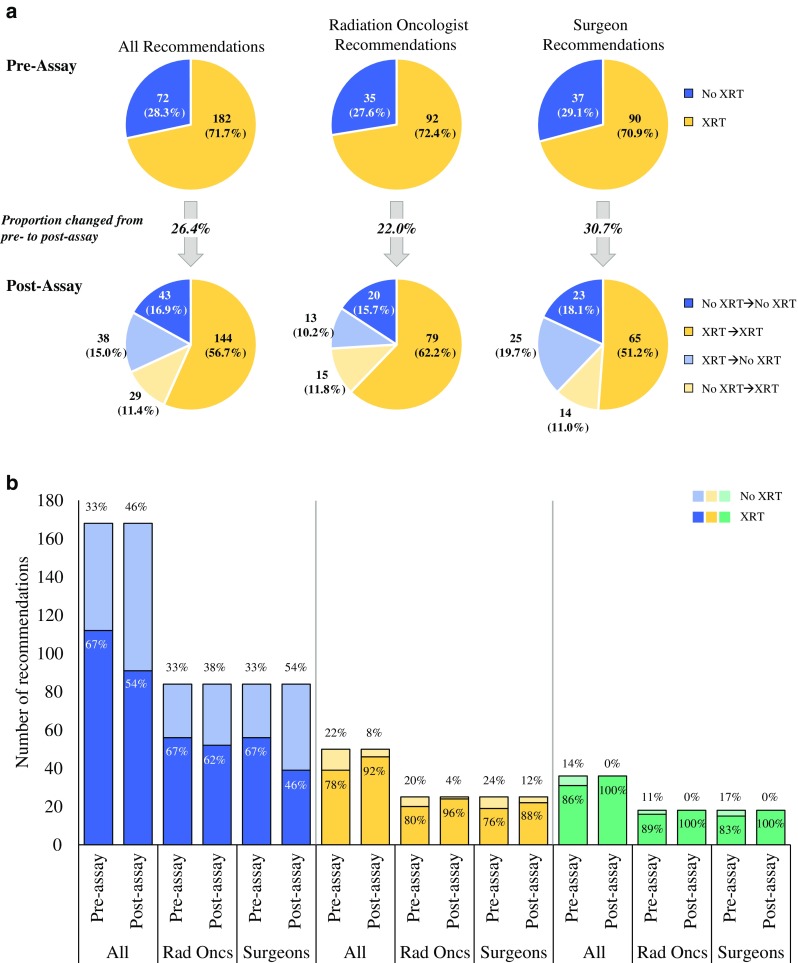
Changes in radiotherapy recommendations from pre- to post-assay by (**a**) specialty and (**b**) DCIS Score risk group. **a** Pre- and post-assay radiotherapy recommendations (combined and by specialty), indicating changes in recommendation, if any, from pre- to post-assay. **b** Pre- and post-assay radiotherapy recommendations (combined and by specialty) made for patients with DCIS Score results in the low (*blue*), intermediate (*yellow*), and high (*green*) ranges. *DCIS* ductal carcinoma in situ, *XRT* radiotherapy, *Rad Oncs* radiation oncologists

Pre-assay, 71.7 % of all recommendations were for radiotherapy, including 72.4 and 70.9 % of recommendations by radiation oncologists and surgeons, respectively (Fig. 3a). Radiation oncologists and surgeons most frequently cited patient age (56.7 and 51.2 %), grade (60.6 and 58.3 %), and size (42.5 and 55.1 %) as factors affecting their pre-assay treatment recommendations. Post-assay, 68.1 % of all recommendations were for radiotherapy, including 74.0 and 62.2 % of recommendations by radiation oncologists and surgeons, respectively (Fig. 3a). Taking into account changes in both directions (from radiotherapy to no radiotherapy, and from no radiotherapy to radiotherapy), the overall change rate was 26.4 %, including 22.0 and 30.7 % for radiation oncologists and surgeons, respectively. Radiation oncologists and surgeons most frequently cited DCIS Score results (61.4 and 69.3 %) as a factor affecting their post-assay treatment recommendations. Radiation oncologists also cited grade (47.2 %) and age (44.9 %), and surgeons also cited size (43.3 %) and age (42.5 %).

Pre- and post-assay radiotherapy recommendations varied by DCIS Score risk group (Fig. 3b). Recommendations for radiotherapy increased for the intermediate and high DCIS Score groups. Notably, all patients with high DCIS Score results had post-assay recommendations for radiotherapy from both specialties. For the low DCIS Score group, recommendations for radiotherapy decreased from pre- to post-assay overall, although the reduction was greater for surgeons (from 67 to 46 %) than for radiation oncologists (from 67 to 62 %).

### Physician Estimates of Local Recurrence Risk, Pre- and Post-Assay

Pre-assay risk estimates were similar between specialties (electronic supplementary Fig. 1). Pre-assay risk estimates by both radiation oncologists and surgeons varied widely, even within DCIS Score groups. For all patients, post-assay risk estimated by radiation oncologists was higher than the risk estimated by surgeons. For the high DCIS Score group, risk estimates by both specialties increased substantially from pre- to post-assay (electronic supplementary Fig. 1).

### Patient-Reported Outcomes

Thirty-two patients (25.2 %) completed the DCS and STAI questionnaires (Table 2). Their decisional conflict decreased significantly from pre- to post-assay, regardless of their DCIS Score risk category (*p* = 0.004) (Table 2). Both state anxiety (*p* = 0.042) and trait anxiety (*p* = 0.047) also decreased significantly.

**Table 2:** Decisional conflict scale and state-trait anxiety inventory, before and after availability of DCIS Score results (pre- and post-assay; *n* = 32)

	Mean score (SD)		Mean change^a^ (95 % CI)	*p* Value
	Pre-assay	Post-assay		
Decisional conflict scale^b^	47.0 (17.9)	37.7 (15.4)	9.2 (3.2–15.2)	0.004
State-trait anxiety inventory				
S-anxiety score^b^	36.8 (11.4)	32.9 (11.7)	3.9 (0.15–7.6)	0.042
T-anxiety score^b^	34.5 (8.9)	32.2 (8.9)	2.3 (0.03–4.53)	0.047

Patients with confirmed and completed questionnaires

*CI* confidence interval, *DCIS* ductal carcinoma in situ, *SD* standard deviation, *S*-*Anxiety* state anxiety (anxiety induced temporarily by situations perceived as dangerous), *T*-*Anxiety* trait anxiety (relatively enduring disposition to feel stress, worry, and discomfort)

^a^ Mean difference from pre- to post-assay

^b^ A lower score represents a lower level of decisional conflict or anxiety; a higher score represents a greater level of decisional conflict or anxiety

## Discussion

The results of this study corroborate findings of an earlier clinical utility study, shed new light on how DCIS Score results are used by radiation oncologists and surgeons, and show how the assay impacts patients, as measured by patient-reported outcomes. The pre-assay radiotherapy recommendation rate of >70 % that we observed is consistent with current US practice and reflects a propensity to treat patients diagnosed with DCIS with radiotherapy.^23^ We show that DCIS Score results changed radiotherapy recommendations 26.4 % of the time, which is consistent with the 31.3 % rate of change reported previously.^17^ Physicians cited DCIS Score results more than any other factor as influencing their post-assay treatment recommendations. Because clinical utility of an assay is defined in part by its capacity to affect treatment decisions,^24^,^25^ our study findings support the clinical utility of the DCIS Score assay. Our findings show that physicians value the point estimates of risk provided by DCIS Score results and integrate them into their overall risk assessments to determine, for each patient, whether or not to recommend radiotherapy.

Our study was designed to assess how DCIS Score results affect treatment recommendations made by both surgeons and radiation oncologists. Notably, the rate of change in recommendations was lower for radiation oncologists than for surgeons, affecting, in particular, patients with low DCIS Score results. Potential explanations for this observation include the possibilities that radiation oncologists may use the DCIS Score risk estimates more, and surgeons may use the risk categories more (low, intermediate, high); that radiation oncologists are generally less likely than surgeons to withhold radiotherapy, even for low-risk patients; or that radiation oncologists and surgeons both incorporate genomic information into their risk assessment algorithms but may give different weights to clinicopathologic factors, such as tumor size and patient age, which are known to be prognostic of local recurrence. We observed that physicians use DCIS Score-associated risk estimates along with clinicopathologic factors to augment risk estimates, in accordance with previously published data.^16^ In certain cases, physicians made treatment recommendations based on the DCIS Score risk estimates, not necessarily the DCIS Score risk group, suggesting that physicians use risk estimates provided by DCIS Score results to individualize patient treatment, even among patients within the same risk group. To better understand the value of the DCIS Score result as a continuous or categorical variable to estimate risk, further investigation would be needed.

The baseline patient and tumor characteristics of our cohort closely matched those of the earlier clinical utility study and the Ontario validation study. Therefore, our cohort is generally representative of patients seen in contemporary clinical practice. Nonetheless, pre-assay median risk estimates by physicians in our study (14 % by both specialties) were lower than that previously reported (20 %), suggesting possible bias in selecting patients with ‘low-risk’ characteristics in whom physicians would feel comfortable recommending to withhold radiation or perhaps changes in perceptions of risk that a DCIS diagnosis might represent. As a survey-based study, we could not control how patients were selected for participation, and the study was not designed to adjust for that bias in the analysis.

Although considerable proportions of patients in our study had clinicopathologic features associated with poorer prognosis (e.g. 40 % with tumors >1 cm in size, 30 % with high-grade tumors, 55 % with margin widths ≤3 mm), DCIS Score results varied substantially across the entire cohort, as well as within the subgroups defined by specific clinicopathologic features. This indicates that DCIS Score results provided information about local recurrence risk beyond what can be gleaned from the clinicopathologic factors alone, and that is based on the individual patient’s underlying tumor biology. Based on previous trials that detected no significant changes in disease-specific or overall survival with or without radiotherapy after BCS, the appropriateness of using survival outcomes as markers of DCIS treatment success is debatable. By shifting the focus to local recurrence, the assessment of individual treatment plans based on disease-specific biology can then be more closely correlated to outcome.

Our study is the first to report how the use of a genomic assay affects decision making and attitudes of patients with DCIS. The pre-assay mean DCS score suggested that patients had feelings of delay in decision making and uncertainty about implementation.^26^ Findings suggest that the results of the DCIS Score assay may have reduced decisional conflict for patients. With respect to anxiety, the pre-assay mean STAI scores suggested that these patients were not overly anxious at baseline.^27^–^29^ Both state and trait anxiety were reduced post-assay. The fact that the mean difference in state anxiety was greater suggests that state anxiety was reduced more than trait anxiety. Since participation in this part of the study was optional, the number of patients with confirmed completed questionnaires was relatively small. Thus, further research is needed to better understand the impact of genomic assays on patient anxiety and decisional conflict.

## Conclusions

DCIS Score results provide individualized, quantitative estimates of local recurrence risk that are based on underlying tumor biology, information that is not always apparent from clinicopathologic features. While an association between the DCIS Score assay and benefit from radiotherapy has not yet been shown, an initial analysis of the patient cohort in the Ontario study that had BCS plus radiotherapy showed a proportional reduction in local recurrence that translates to a smaller absolute benefit of radiotherapy for patients with low DCIS Score results relative to the absolute benefit for patients with high DCIS Score results.^30^ Further investigation is needed to confirm these initial observations. Ongoing work includes the evaluation of additional genes that may provide more information regarding sensitivity to radiotherapy, as well as assessment of the DCIS Score result in a contemporary cohort, to take into consideration the dramatic improvements in recurrence outcomes realized in the last decade. Nevertheless, the DCIS Score assay can be used in conjunction with clinicopathologic features in a more individualized approach to personalizing recommendations for radiotherapy. This approach to patient management is an important first step towards addressing the issue of over- and undertreatment in patients with DCIS.

## Electronic supplementary material

Below is the link to the electronic supplementary material.

Supplementary material 1 (PDF 1231 kb)
