# Supplementary material for: Clinical Utility of the 12-Gene DCIS Score Assay: Impact on Radiotherapy Recommendations for Patients with Ductal Carcinoma In Situ
Source: Ann Surg Oncol. 2016 Oct 4;24(3):660–8. doi: 10.1245/s10434-016-5583-7 (PMC5306072; doi:10.1245/s10434-016-5583-7)
Supplement: Supplementary file 1 — Supplementary material 1 (PDF 1231 kb) [file 10434_2016_5583_MOESM1_ESM.pdf]

Supplemental Materials

- **Supplemental Figure 1**
- **Pre-assay Questionnaire**
- **Post-assay Questionnaire**

Supplemental Figure 1. Pre- and post-assay estimates of 10-year local recurrence risk made by (a) radiation oncologists and by (b) surgeons, for all patients and patients by DCIS Score risk group. Pre- and post-assay mean (standard deviation) and median (range) estimates are shown in tables alongside graphs. Each line in each graph represents one physician's estimate from pre- to post-assay.

a.

Each line represents one radiation oncologist's recomendation

All patients (N=127)

Pre	Mean 15.8 (8.6)<br>Median 14 (4-50)
Post	Mean 16.3 (8.1)<br>Median 15 (1-50)

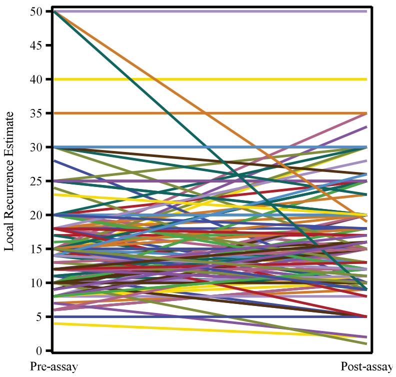

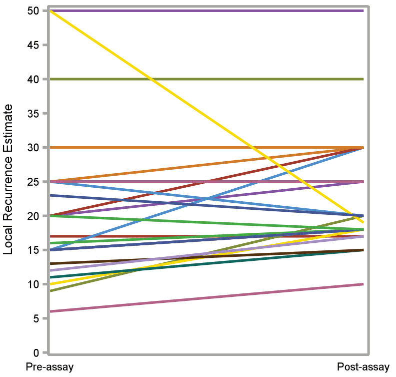

DCIS Intermediate (n=25)

Pre	Mean 21.3 (11.4)<br>Median 20 (6-50)
Post	Mean 22.8<br>Median 20 (10-50)

DCIS Low (n=84)

Pre	Mean 13.7 (6.9)<br>Median 12 (4-50)
Post	Mean 12.2 (4.8)<br>Median 11.5 (1-35)

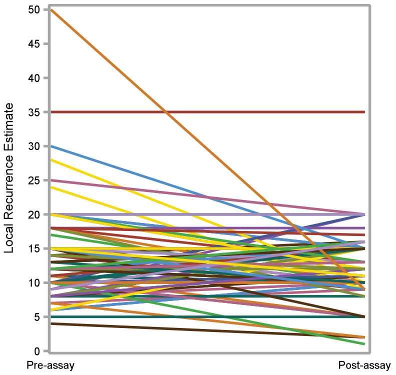

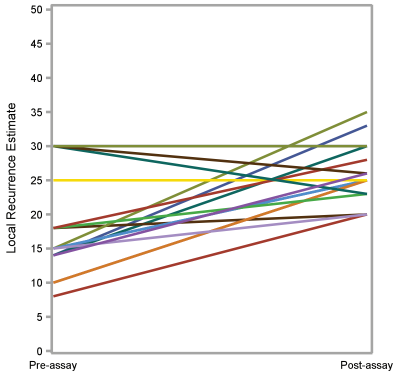

DCIS High (n=18)

Pre	Mean 18.3 (7.4)<br>Median 15 (8-30)
Post	Mean 26.1 (4.3)<br>Median 25 (20-35)

b.

Each line represents one suregon's recomendation

All patients (N=127)

Pre	Mean 14.7 (7.7)<br>Median 14 (5-40)
Post	Mean 14.5 (7.4)<br>Median 14 (0-40)

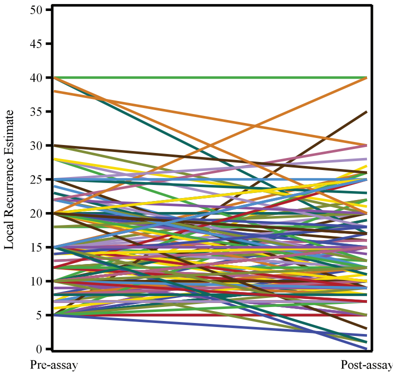

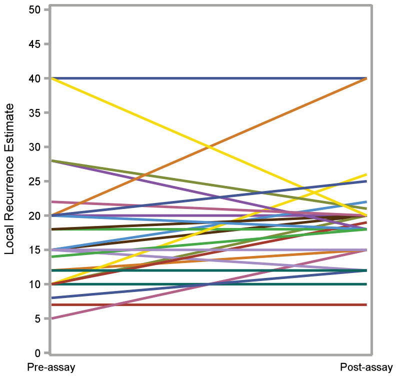

DCIS Intermediate (n=25)

Pre	Mean 17.3 (9.0)<br>Median 15 (5-40)
Post	Mean 19.3 (7.7)<br>Median 19 (7-40)

DCIS Low (n=84)

Pre	Mean 13.3 (6.5)<br>Median 12 (5-40)
Post	Mean 11.0 (4.6)<br>Median 10 (0-25)

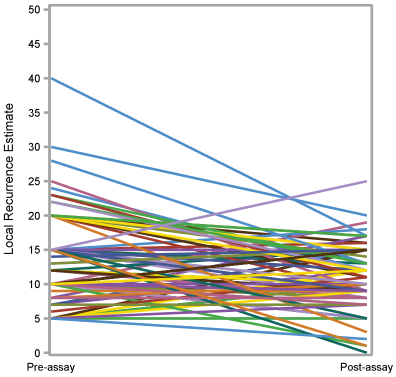

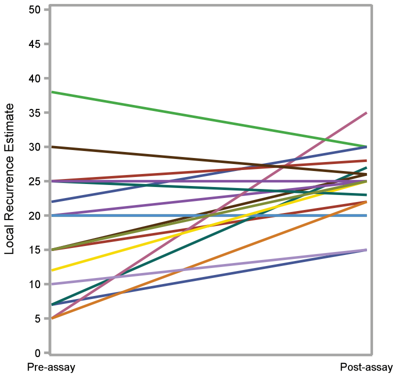

DCIS High (n=18)

Pre	Mean 17.6 (9.1)<br>Median 17.5 (5-38)
Post	Mean 24.4 (5.0)<br>Median 25 (15-35)

**PRE-DCIS SCORE QUESTIONNAIRE**
**Patient ID Number:** [ ][ ][ ][ ]<br><br>**Visit Date:** [ ][ ] — [ ][ ][ ] — [ ][ ][ ][ ]<br>D D M M M Y Y Y Y<br>**Physician ID:** ____________________________________<br>(Physician's **First Initial** combined with their **Last Name** using all lowercase letters. Ex: Dr. John Doe would use jdoe)<br>**Physician:** □ **Surgeon** □ **Radiation Oncologist**
**1. What is your estimate of this patient's 10-year risk of local (same breast) recurrence? (Please enter a percentage)**
_____ %
**2. Would you recommend radiation treatment for this patient right now?**
□ Yes<br>□ No
**3. Please identify up to three of the top reasons for your treatment recommendation**
□ Patient age<br>□ Tumor grade<br>□ Tumor size<br>□ Margins<br>□ Comedonecrosis<br>□ Patient preference<br>□ Patient co-morbidities<br>□ Other pathology measures (please specify):<br>____<br><br>□ Other reason (please specify):<br>____

PHYSICIAN POST-DCIS SCORE QUESTIONNAIRE

Patient ID Number: ☐☐☐☐

Visit Date: ☐☐ __ ☐☐☐ __ ☐☐☐☐
D D M M M Y Y Y Y

Physician ID: ____________________________________
(Physician's **First Initial** combined with their **Last Name** using all lowercase letters. Ex: Dr. John Doe would use jdoe)

Physician: ◻ **Surgeon** ◻ **Radiation Oncologist**

Requisition Number: ______________________

1. DCIS Score™: _________

2. What is your estimate of this patient's 10-year risk of local (same breast) recurrence? (Please enter a percentage)

______ %

3. Would you recommend radiation treatment for this patient right now?

◻ Yes
◻ No

4. Please identify up to three of the top reasons for your treatment recommendation

◻ Patient age
◻ Tumor grade
◻ Tumor size
◻ Margins
◻ Comedonecrosis
◻ Patient preference
◻ Patient co-morbidities
◻ Other pathology measures (please specify):
____

◻ DCIS Score Result
◻ Other reason (please specify):
____

5. Was the Onco*type* DX assay useful for this patient?

◻ Yes
◻ No
Reason (Why/Why not?):
____
